# Annual consultation prevalence of regional musculoskeletal problems in primary care: an observational study

**DOI:** 10.1186/1471-2474-11-144

**Published:** 2010-07-02

**Authors:** Kelvin P Jordan, Umesh T Kadam, Richard Hayward, Mark Porcheret, Catherine Young, Peter Croft

**Affiliations:** 1 Arthritis Research UK Primary Care Centre, Primary Care Sciences, Keele University, Keele, Staffs ST5 5BG UK

## Abstract

**Background:**

Regional musculoskeletal pain such as back or shoulder pain are commonly reported symptoms in the community. The extent of consultation to primary care with such problems is unknown as a variety of labels may be used to record such consultations. The objective was to classify musculoskeletal morbidity codes used in routine primary care by body region, and to determine the annual consultation prevalence of regional musculoskeletal problems.

**Methods:**

Musculoskeletal codes within the Read morbidity Code system were identified and grouped by relevant body region by four GPs. Consultations with these codes were then extracted from the recorded consultations at twelve general practices contributing to a general practice consultation database (CiPCA). Annual consultation prevalence per 10,000 registered persons for the year 2006 was determined, stratified by age and gender, for problems in individual regions and for problems affecting multiple regions.

**Results:**

5,908 musculoskeletal codes were grouped into regions. One in seven of all recorded consultations were for a musculoskeletal problem. The back was the most common individual region recorded (591 people consulting per 10,000 registered persons), followed by the knee (324/10,000). In children, the foot was the most common region. Different age and gender trends were apparent across body regions although women generally had higher consultation rates. The annual consultation-based prevalence for problems encompassing more than one region was 556 people consulting per 10,000 registered persons and increased in older people and in females.

**Conclusions:**

There is an extensive and varied regional musculoskeletal workload in primary care. Musculoskeletal problems are a major constituent of general practice. The output from this study can be used as a resource for planning future studies.

## Background

In the UK, primary care is commonly the point of entry into the health care system for people with a new symptom or illness and the major source of continuing care for chronic conditions. Musculoskeletal problems are one of the most common reasons for seeking primary care, with estimates of up to 20% of adults consulting their general practitioner with a musculoskeletal problem over the course of a year[1,2].

In order to plan primary health care and monitor the clinical course of problems presented to primary care, increasing use is made of routine electronic recording of the reasons people consult. There is much interest in estimating the level of health care need for musculoskeletal disorders, and then in identifying suitable and effective interventions. Patients with musculoskeletal problems often present to primary care with a regional symptom, such as back, knee or shoulder pain. Many musculoskeletal symptoms cannot be labeled with a diagnosis initially and GPs often find it more useful to work with a regional pain label than a complex diagnostic label. Region specific management is common in primary care[3]. Randomised controlled trials in primary care of management of regional pain symptoms, as opposed to diagnoses, are increasing (e.g. [4-6]), leading to requirements to estimate the number of people who consult who meet the criteria for recruitment. Epidemiological studies are increasingly using routinely recorded primary care data to model occurrence, management and outcome of consultation for regional and widespread musculoskeletal problems, and so also need to identify patients with specific regional problems[7-10].

Generally, the increasing use and quality of electronic records in primary care means that it should be easier to determine the consultation prevalence related to specific types of morbidity. UK databases such as the Fourth National Survey of Morbidity in General Practice (MSGP4) conducted by the Royal College of General Practitioners (RCGP) last carried out in 1991/2 [11] and the linked annual RCGP Weekly Returns Service (WRS) reports [2] provide figures on annual prevalence of consultation to primary care. However, in terms of musculoskeletal disorders, these problems are often either given diagnostic labels such as osteoarthritis or rheumatoid arthritis, or labelled as non-specific disorders. Systems of morbidity coding do not lend themselves easily to the identification of prevalence of consultation for individual regional presenting symptoms, because the principles of coding in primary care do not neatly or necessarily reflect a regional classification of symptoms. The Read Code hierarchy, a common system for recording morbidity in UK primary care, is structured into diagnostic chapters (for example, Musculoskeletal diseases, Mental disorders, Circulatory disorders)[12]. The most common musculoskeletal problems diagnosed in primary care by the RCGP WRS in 2007 within the Musculoskeletal Read Code Chapter were listed as "Back disorders unspecified other", "Pain in Limb", "Joint Disorder Other Unspecified", "Osteoarthritis & Allied Disorders" and "Peripheral Enthesopathies & Allied Syndromes"[2].

Most musculoskeletal morbidity codes fall within the Musculoskeletal Disorders Chapter of coding systems but may also fall into the symptoms Chapters or the injury Chapter. The Read Code hierarchy has, for example, over a hundred knee problem codes under different diagnostic chapters[13]. Widespread or generalised problems are often labelled as multiple individual problems in primary care[10], and labels such as fibromyalgia are rarely used[14]. Previous studies of regional problems in primary care which have used general practice consultation databases have been limited by either concentrating on a subset of codes (generally from within the Musculoskeletal Disorders Chapter), had small samples, have scrutinised written records, or restricted analysis to specific sites or ages[9,15-19]. They have generally not allowed comparison of the relative prevalence of primary care consultation across multiple body regions and therefore the extent to which presentation of musculoskeletal problems by region varies is unknown.

The aims of this study were to determine the annual consultation prevalence for musculoskeletal problems in primary care, classified by body region, and to develop a resource for primary care researchers working in this field.

## Methods

The study was performed by first identifying all musculoskeletal Read Codes and classifying them into body regions, and then applying the classification to a primary care consultation database.

### Setting

The setting was the Consultations in Primary Care Archive (CiPCA), a high quality, validated database, which contains all recorded consultation data from a subset of general practices in North Staffordshire, UK, since 1998[1,20]. Ethical approval for CiPCA was granted by the North Staffordshire Research Ethics Committee. In 2006, twelve practices contributed to CiPCA. These practices cover a range of areas in terms of deprivation although generally North Staffordshire is more deprived than England as a whole. In the UK, over 95% of the population are registered with a general practice and general practice is the first point of access to the National Health Service for most non-emergency care in the UK.

The practices undergo an annual cycle of assessment, feedback and training in morbidity coding[20]. Doctors and nurses are requested to enter at least one morbidity code for each contact. Whilst encouraged to use diagnostic codes, symptom codes may be used until a diagnosis is made. In 2006, 97% of all contacts with a GP had a morbidity code assigned.

CiPCA has been shown to give similar annual consultation prevalence rates for musculoskeletal problems (defined as codes under Chapter N "Musculoskeletal and connective tissue diseases" of the Read Code hierarchy), osteoarthritis, rheumatoid arthritis, and arthralgia as the larger, national RCGP WRS[1].

### Identification of codes and allocation to regions

In the first stage of the study, two GPs independently identified all morbidity Read codes potentially relating to pain or musculoskeletal disorders, irrespective of Read Code Chapter assignment. Agreement on the framework for allocation of codes to regions and formal training in its use were performed using a sample of 100 codes. Four GPs allocated the full set of codes to individual body regions (e.g. back, knee). For the purposes of this study, all identified Codes under Chapters N "Musculoskeletal and connective tissues diseases", R "Symptoms, Signs and Ill-defined conditions", S "Injury and Poisoning" and 1 "History/Symptoms" were used as they were deemed to be predominantly musculoskeletal. If no body region could be allocated, the code was defined as "unspecified". "Unspecified" problems tended to be codes where either no region was described in the associated Read Term (e.g. the term simply specified "arthralgia") or the problem covered more than one region (e.g. "generalised osteoarthritis"). For codes which were labelled as unspecified, a research GP and a non-clinical researcher further identified those defining a "more widespread" or generalised problem (i.e. likely to cover more than one body region) based on the associated Read term. Examples of widespread terms include terms such as "generalised osteoarthritis" and "multiple arthralgia". The list of Read Codes and associated terms relating to the knee, and those relating to generalised or widespread problems, are given as examples of the classification in additional file 1.

The defined regions were then grouped into four main body sectors: (i) head/neck, (ii) torso, (iii) upper limb, and (iv) lower limb. Regions covered by the torso sector included the back (upper, lower, or not stated), abdomen, chest, and trunk. The upper limb sector included consultations for hand, wrist, elbow, forearm, shoulder, and upper arm. The lower limb sector included consultations specified as foot, ankle, lower leg, knee, thigh, hip, and pelvis.

### Measurement of annual consultation prevalence

For the calendar year 2006, all consultations at the surgery, home visit, or by telephone which had one of the identified morbidity codes were extracted. Some codes have alternative terms (for example, Read Code N05z6 has the terms "osteoarthritis of the lower leg" and "osteoarthritis of the knee"). Therefore final checks of the terms were made to ensure the correct region was allocated based on the term as well as the code (for example, allocation to lower leg or knee).

"Annual consultation prevalence" was defined as the proportion of all people registered with the practices who had consulted at least once in the year with the relevant problem. People could only be counted once for any region or sector even if they consulted for more than one treatment episode or problem in the same region or sector. Hence all further consultations for the same region (or sector as appropriate) following the first consultation for that region/sector in the year were ignored.

Using the number of people registered at the practices at mid-year (1^st ^July) as the denominator, annual consultation prevalence figures per 10,000 registered persons, stratified by age and gender, were determined for each region of the body, and for each sector (head/neck, torso, upper and lower limb). A total population standardised consultation prevalence rate was also determined for each region, using direct standardisation, by applying the CiPCA prevalence rates to the age-gender general population structure of England and Wales in 2006[21].

Differences between genders in regional prevalence rates were assessed through use of negative binomial regression, adjusting for age group. The female to male prevalence rate ratio with 95% confidence interval is reported.

The annual consultation-based prevalence of "widespread" problems was also determined. This was based on two definitions: i) separate recorded consultations for regions in two or more of the four sectors (head/neck, torso, upper limb, lower limb); ii) a recorded generalised/widespread code during the year based on the codes given in the additional file 1.

### Region and sector consultations as percentage of all musculoskeletal consultations

Finally, as a measure of relative workload related to individual regions and sectors, the total number of consultations (including repeat consultations) for each region, and for each sector, was calculated as a percentage of all musculoskeletal consultations.

Analysis was performed using SPSS 15.0 for Windows and Stata/IC 10.0 for Windows.

## Results

### Identification of codes and allocation to regions

5,908 morbidity Read codes were identified by the research GPs as being musculoskeletal in nature. 4,862 (82%) of these were allocated by these GPs to a region of the body, the remainder were defined as unspecified. In 2006, the total registered population of the practices was 100,758 persons. During the course of 2006, there were 55,033 consultations which had been recorded with one of the 5,908 musculoskeletal codes. 47,359 (86%) of these could be allocated to a body region, 4,322 (8%) were defined as a generalised/widespread problem, and the remainder had no region specified. Read Codes from the Musculoskeletal Disorders Chapter (Chapter N) were used in 73% of musculoskeletal consultations (ranging from 52% of musculoskeletal consultations in those aged under 15 to 79% of musculoskeletal consultations in those aged 65-74).

### Any musculoskeletal problem

Musculoskeletal problems, including repeat consultations, accounted for 14% of all consultations that received a diagnostic or symptom code at the practices. This ranged from 4% of all consultations for those aged under 15, to 17% of all consultations in those aged 45-64.

A quarter of the registered population consulted at least once with a musculoskeletal problem during the course of the year (2405 per 10,000 persons; 95% CI 2380, 2430) (tables 1, 2). This rate increased with age and was higher in females (female to male prevalence rate ratio 1.13; 95% CI 1.05, 1.22). A similar trend was seen when restricting the analysis to codes within the Musculoskeletal Disorders Chapter (Chapter N).

**Table 1:** Annual consultation prevalence per 10,000 registered persons for any musculoskeletal problem and by sector - total, and males by age group

	Total^a^	Male (age group)					
		0-14	15-24	25-44	45-64	65-74	75+
Any musculoskeletal	2405	756	1553	2235	2837	3277	3339
Chapter N only	1803	373	799	1554	2200	2659	2700
Sectors							
Head/neck	298	145	201	259	274	348	338
Torso	843	134	536	923	1054	1001	1051
Upper limb	498	85	301	471	696	729	585
Lower limb	1010	360	562	824	1181	1553	1642

^a ^males and females; age-gender standardised based on population figures for England and Wales in 2006[21]

**Table 2:** Annual consultation prevalence per 10,000 registered persons for any musculoskeletal problem and by sector - total, and females by age group

	Total^a^	Female (age group)					
		0-14	15-24	25-44	45-64	65-74	75+
Any musculoskeletal	2405	597	1801	2565	3515	3958	4149
Chapter N only	1803	339	1156	1910	2886	3392	3337
Sectors							
Head/neck	298	113	257	416	425	440	416
Torso	843	106	648	1029	1212	1321	1246
Upper limb	498	84	291	517	865	692	730
Lower limb	1010	267	693	924	1486	1933	2096

^a ^males and females; age-gender standardised based on population figures for England and Wales in 2006[21]

### Prevalence by sector

Annual consultation prevalence for any part of the lower limb increased with age and was slightly higher for females (rate ratio 1.16; 95% CI 1.07, 1.26). Consultation prevalence for any part of the upper limb increased with age to 45-64 and then stabilised, and was also slightly higher in females (rate ratio 1.12; 95% CI 1.04, 1.20) (tables 1,2).

### Regional problems

Table 3 shows the age-gender standardised consultation prevalences for the most common body regions, with the age and gender specific rates for all regions in tables 4, 5, 6 and 7. The back, and predominantly the lower back, was the most common problem site for all age groups except for children where the foot was the most common. Consultation prevalence increased consistently with age for some regions (for example, neck, knee, hip), others showed a sharp increase in consultation prevalence around age groups 25-44 or 45-64 (for example, chest, back, shoulder, foot, ankle). Other patterns of note are the increased consultation prevalence for the elbow in those aged 45-64, the decreasing consultation prevalence for wrist problems in adults as age increases, and the higher consultation prevalence for pelvic problems in females aged 25-44. Females had a higher consultation prevalence in general and, in particular for the hip (rate ratio 1.64; 95% CI 1.46, 1.85), wrist (1.59; 95% CI 1.34, 1.88) and neck (1.44; 95% CI 1.28, 1.63), and the less common sites of upper back (1.90; 95% CI 1.43, 2.52), forearm (1.90; 95% CI 1.15, 3.13) and upper arm (2.07; 95% CI 1.40, 3.05), but not for the elbow (0.75; 95% CI 0.55, 1.01).

**Table 3:** Annual consultation prevalence and gender rate ratio for the most common regional problems

Region	**Rate**^**a **^**per 10,000 persons**	(95% CI)	Female:male rate ratio (95% CI)
Back (any^b^)	591	(577, 606)	1.22 (1.14, 1.31)
Lower back	417	(405, 429)	1.20 (1.13, 1.28)
Knee	324	(313, 334)	1.03 (0.93, 1.15)
Chest	280	(270, 290)	1.04 (0.97, 1.12)
Neck	228	(219, 237)	1.44 (1.28, 1.63)
Foot	208	(200, 217)	1.18 (1.05, 1.33)
Shoulder	199	(191, 207)	1.11 (1.02, 1.21)
Hand	132	(125, 139)	1.05 (0.86, 1.28)
Hip	115	(108, 121)	1.64 (1.46, 1.85)
Pelvis.	100	(94, 106)	1.17 (0.98, 1.41)
Ankle.	88	(82, 94)	1.10 (0.94, 1.29)
Head	86	(81, 92)	1.29 (1.08, 1.55)
Elbow	78	(73, 84)	0.75 (0.55, 1.01)
Wrist	58	(53, 62)	1.59 (1.34, 1.88)

^a ^males and females; age-gender standardised based on population figures for England and Wales in 2006[21]

^b ^includes consultations coded as upper back, lower back, or back

**Table 4:** Annual consultation prevalence per 10,000 registered persons for regions in the head/neck and torso - total, and males by age group

	Total^a^	Male (age group)					
		0-14	15-24	25-44	45-64	65-74	75+
Head	86	105	80	70	59	72	64
Neck	228	59	131	196	224	291	293
Abdomen	4	1	3	4	4	4	12
Chest	280	70	198	269	380	421	402
Lower back	417	30	209	487	536	486	448
Upper back	21	2	8	17	18	20	24
Back (any^b^)	591	63	336	680	726	644	661
Trunk	2	1	10	1	1	2	6

^a ^males and females; age-gender standardised based on population figures for England and Wales in 2006[21]

^b ^includes consultations coded as upper back, lower back, or back

**Table 5:** Annual consultation prevalence per 10,000 registered persons for regions in the head/neck and torso - total, and females by age group

	Total^a^	Female (age group)					
		0-14	15-24	25-44	45-64	65-74	75+
Head	86	78	105	107	105	72	97
Neck	228	52	174	334	346	383	336
Abdomen	4	0	3	5	4	6	2
Chest	280	43	200	288	409	450	398
Lower back	417	29	286	529	634	677	611
Upper back	21	8	22	27	28	50	60
Back (any^b^)	591	65	463	767	845	930	898
Trunk	2	0	2	1	2	0	4

^a ^males and females; age-gender standardised based on population figures for England and Wales in 2006[21]

^b ^includes consultations coded as upper back, lower back, or back

**Table 6:** Annual consultation prevalence per 10,000 registered persons for regions in the upper and lower limbs - total, and males by age group

	Total^a^	Male (age group)					
		0-14	15-24	25-44	45-64	65-74	75+
Elbow	78	6	23	98	162	90	101
Forearm	7	1	3	8	4	2	6
Hand	132	46	144	122	142	177	134
Shoulder	199	7	59	177	312	366	280
Upper arm	11	0	5	9	10	7	15
Wrist	58	12	64	54	52	39	27
Ankle	88	36	69	103	94	90	110
Foot	208	107	90	163	239	315	329
Hip	115	23	38	38	111	241	232
Knee	324	85	209	288	415	519	597
Lower leg	41	33	41	36	42	68	73
Pelvis	100	26	73	99	108	145	113
Thigh	19	9	20	11	22	24	24

^a ^males and females; age-gender standardised based on population figures for England and Wales in 2006[21]

**Table 7:** Annual consultation prevalence per 10,000 registered persons for regions in the upper and lower limbs - total, and females by age group

	Total^a^	Female (age group)					
		0-14	15-24	25-44	45-64	65-74	75+
Elbow	78	3	15	86	152	42	38
Forearm	7	4	3	7	14	8	16
Hand	132	34	94	127	242	166	164
Shoulder	199	9	73	184	347	398	343
Upper arm	11	3	7	14	27	13	27
Wrist	58	18	97	83	83	48	80
Ankle	88	27	77	85	135	122	117
Foot	208	90	132	194	356	341	316
Hip	115	33	57	75	184	358	400
Knee	324	54	192	242	472	667	686
Lower leg	41	13	25	40	41	55	93
Pelvis	100	24	134	158	101	133	100
Thigh	19	1	3	17	33	29	51

^a ^males and females; age-gender standardised based on population figures for England and Wales in 2006[21]

### Widespread problems

Widespread problems defined as separate codes for regional problems in different sectors of the body (recorded in 405 per 10,000 persons) were more common than a recording of a widespread or generalised code (177 per 10,000 persons). Annual consultation prevalence for widespread problems (defined in either way) was 556 per 10,000 persons, ranging from 51 per 10,000 females aged 14 or under to 1388 per 10,000 females aged 65-74 (tables 8, 9). There was a strong age trend (*p *< 0.001) and a higher prevalence in females (rate ratio 1.57; 95% CI 1.48, 1.67).

**Table 8:** Annual consultation prevalence per 10,000 registered persons for widespread problems - total, and males by age group

	Total^a^	Male (age group)					
		0-14	15-24	25-44	45-64	65-74	75+
2 sectors^b^	361	39	124	309	441	550	521
3-4 sectors^b^	44	0	13	23	60	68	58
Generalised code^c^	177	12	26	50	135	250	341
Total^d^	556	52	162	378	621	837	887

^a ^males and females; age-gender standardised based on population figures for England and Wales in 2006[21]

^b ^individual codes for regions in different sectors

^c ^single code referring to problems in more than one region

^d ^consultation for widespread musculoskeletal problems defined as either individual codes for regions in different sectors or a generalised code

**Table 9:** Annual consultation prevalence per 10,000 registered persons for widespread problems - total, and females by age group

	Total^a^	Female (age group)					
		0-14	15-24	25-44	45-64	65-74	75+
2 sectors^b^	361	32	200	378	624	728	735
3-4 sectors^b^	44	1	5	49	100	103	71
Generalised code^c^	177	18	57	110	384	673	673
Total^d^	556	51	256	521	1049	1388	1370

^a ^males and females; age-gender standardised based on population figures for England and Wales in 2006[21]

^b ^individual codes for regions in different sectors

^c ^single code referring to problems in more than one region

^d ^consultation for widespread musculoskeletal problems defined as either individual codes for regions in different sectors or a generalised code

### Region and sector consultations as percentage of all musculoskeletal consultations

**Figure 1:**
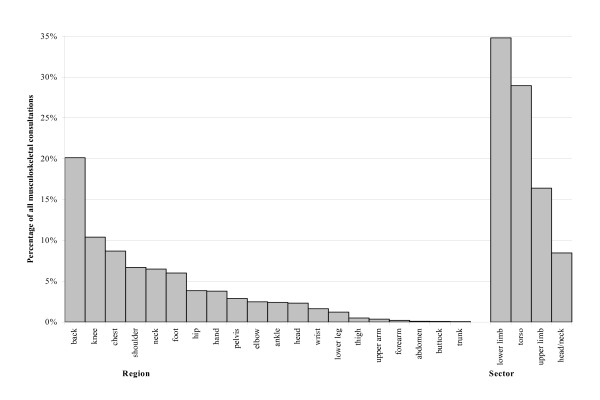
Regional consultations as percentage of all musculoskeletal consultations

One in three of all musculoskeletal consultations were for the lower limb (figure 1). The torso accounted for 29% of all musculoskeletal consultations and the upper limb accounted for 16%. The back was the most common region seen in primary care accounting for 20% of all musculoskeletal consultations (the lower back specifically accounted for 14%). The knee was the next most common regional problem seen, being recorded in 10% of all musculoskeletal consultations. The one exception was for children under 15 years where the foot was the most commonly recorded region (14% of all musculoskeletal problems recorded).

## Discussion

This study has highlighted the frequency of musculoskeletal problems in primary care. One in seven of all recorded consultations in the twelve study practices during 2006 was for a musculoskeletal problem. One in four of the registered population consulted for a musculoskeletal problem in that year, rising to more than one in three of older adults. The back was the most common reason for consultation, followed by the knee, chest and neck; except in children where the foot was the predominant regional problem.

Overall consultation prevalence of musculoskeletal problems in adults for codes within the Musculoskeletal Disorders Read Code Chapter was similar to that found in a Swedish study[15]. The finding that females were more likely than males to consult primary care for regional musculoskeletal problems is consistent with previous studies examining consultation prevalence for musculoskeletal symptoms[16], chronic pain[15], lower limb problems[17], shoulder problems[9], and neck and upper limb problems[18]. The one region tending towards male susceptibility (although not statistically significant) was the elbow, which also rose in prevalence in working age groups and may be related to the association of elbow problems with manual occupations.

Other studies have concentrated on specific regions of the body, and have not shown the relative prevalence rates across regions. Consultation prevalence for lower limb problems (10%) in our study was higher than that reported by van der Waal (6%)[17], probably reflecting our inclusion of codes outside of the Read musculoskeletal Chapter. Consultation prevalence for shoulder problems in adults was similar to that (2.4%) reported for adults by Linsell et al who also reported higher prevalence in females and older age groups[9]. Consultations for the shoulder as a proportion of all consultations were also similar to our study (0.71%).

Widespread problems were common with 5% of the registered population consulting for several regions, rising to 10% of those aged 45 and over. Consulting for other regions was seen in a Finnish study in those consulting initially with neck or shoulder problems[22]. There is also evidence from population surveys that multiple site problems dominate in persons with chronic musculoskeletal pain with only a quarter of those reporting chronic pain having it in a single site[23]. That survey also found that females and the older aged were more likely to report multiple pain sites and this is consistent with the consultation patterns we have identified. Although our study was restricted to a period of one year, it suggests a substantial amount of chronic and widespread pain in primary care, a problem known to relate to poorer general health[10]. However, in younger age groups particularly, most encounters are coded as single site problems, and it is only in the older age groups that more generalised codes are recorded by the health care professional.

About one in four musculoskeletal consultations were given codes outside the musculoskeletal (Chapter N) chapter. These included symptoms such as knee pain and injuries such as fractures. Some GPs may prefer to use a symptom code until they can clearly make a diagnosis. Using only codes within the titled musculoskeletal chapter, would lead to an underestimate of the musculoskeletal problems seen in primary care.

This study was performed within a high quality dataset which included practices who undergo annual assessments of their morbidity coding, have reached levels of 97% of contacts being given a code, and give comparable general consultation rates to national databases[1,20]. The grouping of codes to regions was performed by independent clinical practitioners, prior to extraction of consultations from CiPCA. This approach to using morbidity codes to map musculoskeletal regions offers a standardised method to investigate the occurrence, management and outcome of regional problems in other primary care databases using Read codes. Further, alongside our consultation prevalence figures they offer a basis for researchers planning trials and epidemiological studies in primary care.

Our study data was drawn from a registered population of over 100,000 persons but only a small number of practices in one area of UK which is slightly more deprived than England as a whole. However, the CiPCA database has been shown to give comparable age-gender standardised consultation prevalence figures for musculoskeletal problems overall, and for osteoarthritis, rheumatoid arthritis and arthralgia as the larger national RCGP WRS database[1]. Whilst the classification into regions is most relevant to the UK where Read Codes are used, the consultation prevalence figures are likely to be relevant to countries with similar primary care systems.

About 6% of the identified musculoskeletal consultations could not be attributed to a region or to being a widespread problem as the recorded codes lacked enough detail. There is no reason to suggest GPs were less likely to label one region than another but our figures may be a slight underestimate of the true consultation prevalence for individual regional problems. A further issue is the overlapping of regions. For example, overlap has been reported previously in recording of neck and shoulder complaints[19]. Knee pain may also be included in a consultation recorded as lower leg, leading to an underestimate of the consultation prevalence for the knee. However the identified consultation prevalence for lower leg was very low.

It is possible that some of the symptom codes outside of the defined Musculoskeletal Chapter may not have the diagnostic features of musculoskeletal problems. The chest was a common reason for consultation and consultations for chest problems may not always be regarded as a musculoskeletal problem[24]. The predominant chest codes identified in our study was a symptom code labelled "Chest pain" and its daughter code "Musculoskeletal chest pain". It is possible the label of "Chest pain", as well as including musculoskeletal problems, may include problems that are not musculoskeletal. However, it would be expected that if the symptom was cardiac or gastrointestinal in nature, for example, then it would have been coded under these Chapters and hence would not have been identified by our classification, unless the symptom code is being used until a firm diagnosis is made. There is evidence that the main underlying reason for all chest pain seen in primary care is musculoskeletal with separate studies suggesting that musculoskeletal reasons account for between 20% and 49% of chest pain cases[24-26]. This would all suggest the majority of chest pain consultations in our study were musculoskeletal in nature.

We based annual consultation for widespread problems on two different definitions: counts of body sectors from separate consultations and single consultations labelled with a generalised code. Definitions of widespread pain vary and include use of widespread morbidity codes, specific diagnoses such as fibromyalgia, and definitions based on varying numbers and areas of the body. Some criteria are more restrictive than the one we have used[27,28]. This makes comparisons between studies difficult. Our definition indicates people with pain in at least two distinct regions.

As with all studies set within primary care, this study can only measure problems for which health care is sought. The general population prevalence of problems will be higher as many people will have regional pain but do not consult.

## Conclusions

The use of a classification system based on body regions rather than diagnosis highlights the extensive and varied musculoskeletal workload in primary care, and the extent of multiple regional problems. Musculoskeletal problems need to be recognised as a major constituent of general practice, particularly the extent of problems in multiple body regions, highlighted by this new classification system. With the increased use of electronic recording in recent years, this new classification of regional problems provides a potential resource for researchers to standardise randomised controlled trials of regional pain in primary care and identify eligible participants from morbidity records, or to assess the primary care management and outcome of regional musculoskeletal problems.

## Competing interests

The authors declare that they have no competing interests.

## Authors' contributions

KPJ, UTK, MP, CY and PC conceived and designed the study. All authors were involved in carrying out the study and interpreting the results. KPJ performed the statistical analysis and drafted the manuscript. All authors read, commented on and approved the final manuscript.

## Pre-publication history

The pre-publication history for this paper can be accessed here:

http://www.biomedcentral.com/1471-2474/11/144/prepub

## Supplementary Material

Additional file 1 **Read codes and associated terms for the knee region and for generalised/widespread problems**. Read codes and associated terms used to identify knee regional consultations. Read codes and associated terms used to identify generalised/widespread musculoskeletal problems Click here for file
