# Supplementary material for: Annual consultation prevalence of regional musculoskeletal problems in primary care: an observational study
Source: BMC Musculoskelet Disord. 2010 Jul 2;11:144. doi: 10.1186/1471-2474-11-144 (PMC2903510; doi:10.1186/1471-2474-11-144)
Supplement: Additional file 1 — Read codes and associated terms for the knee region and for generalised/widespread problems. Read codes and associated terms used to identify knee regional consultations. Read codes and associated terms used to identify generalised/widespread musculoskeletal problems [file 1471-2474-11-144-S1.DOC]

Read codes and associated terms for the knee region and for generalised / widespread problems

*Knee region*

**Code Term**

14G3 H/O: knee problem

16J4 Swollen knee

1M10 Knee pain

1M12 Anterior knee pain

N01wB Reactive arthropathy of knee

N01zK Infec arthritis NOS-knee

N02zK Crystal arthropathy NOS-knee

N03xB Arthr assoc oth dis-knee

N040D Rheumatoid arthritis of knee

N0536 Patellofemoral osteoarthritis

N05z6 Knee osteoarthritis NOS

N05zL Osteoarthritis NOS of knee

N061M Traumatic arthropathy-knee

N064M Transient arthropathy-knee

N06z6 Knee arthritis NOS

N07 Internal derangement of knee

N070 Medial meniscus derangement

N0700 Medial menisc.derang.unspecif

N0701 Old bucket handle tear-medial

N0702 Medial menisc.ant.horn derang.

N0703 Medial menisc.post.horn derang

N0704 Parr beak tear-post/med menisc

N0705 Periph detach-medial meniscus

N0706 Radial tear of medial meniscus

N0707 Horiz cleavage tear-med menisc

N0708 Multiple tears-medial meniscus

N0709 Cyst of medial meniscus

N070A Old tear of medial meniscus

N070B Old tear post horn med menis

N070z Medial meniscus derangement NOS

N071 Lateral meniscus derangement

N0710 Lateral menisc.derang.unspecif

N0711 Old bucket handle tear-lat men

N0712 Lateral menisc.ant.horn derang

N0713 Lateral menisc.post.horn deran

N0714 Lateral meniscus derangem.NOS

N0715 Parr beak tear-post/lat menisc

N0716 Periph detach-lateral meniscus

N0717 Radial tear-lateral meniscus

N0718 Horiz cleavage tear-lat menisc

N0719 Multiple tears-lat meniscus

N071A Cyst of lateral meniscus

N071B Discoid lateral meniscus

N071C Old tear of lateral meniscus

N072 Meniscus derangement NEC

N0720 Old torn meniscus of knee

N0721 Degen lesion artic cart knee

N0722 Cyst of semilunar cartilage

N073 Loose body in knee

N074 Chondromalacia patellae

N07y Oth. internal knee derangement

N07y0 Old lat.collat.lig.disruption

N07y1 Old med.collat.lig.disruption

N07y2 Old ant.cruciate lig.disrupt.

N07y3 Old post.cruciate lig.disrupt.

N07y4 Old capsular knee lig.disrupt.

N07y5 Locked knee

N07y6 Patellofemoral maltracking

N07y7 Old part tear lat collat lig

N07y8 Old compl tear lat collat lig

N07y9 Old post/lat caps complex tear

N07yA Old part tear med collat lig

N07yB Old compl tear med collat lig

N07yC Old med capsular complex tear

N07yD Old part tear ant cruciate lig

N07yE Old comp tear ant cruciate lig

N07yF Old part tear post cruciat lig

N07yG Old comp tear post cruciat lig

N07yH Locking knee

N07yy Other knee lig. old disruption

N07yz Other intern.knee derang.NOS

N07z Internal knee derangement NOS

N082Q Path disloc-knee joint

N082R Path disloc-patellofem joint

N0836 Patella-recurrent dislocation

N083n Recurrent disloc - knee

N083p Recurrent disloc - patella

N083q Recurrent sublux - patella

N083r Habitual disloc - patella

N0846 Knee joint contracture

N084a Flexion contracture-knee

N085P Ankylosis of the knee joint

N0906 Knee joint effusion

N090M Effusion of knee

N0916 Haemarthrosis of the knee

N091M Haemarthrosis of knee

N092M Villonodular synovitis of knee

N0946 Knee joint pain

N094M Arthralgia of knee

N094W Anterior knee pain

N0956 Knee stiff

N095M Stiff knee NEC

N0966 Knee gives way

N096M Other symptoms - knee

N098B Synov osteochondromat-knee

N099C Clicking knee

N09A Patellofemoral disorder

N09AX Disorder of patella unspecified

N2159 Iliotibial band syndrome

N216 Enthesopathy of knee

N2160 Bursitis of knee NOS

N2162 Tibial collateral lig.bursitis

N2163 Fibular collat.lig.bursitis

N2164 Patellar tendinitis

N2165 Prepatellar bursitis

N2166 Infrapatellar bursitis

N2167 Subpatellar bursitis

N216z Suprapatellar bursitis

N220z Synovitis of knee

N2222 Beat knee

N2224 Miners' knee

N2225 Housemaids knee

N2246 Ganglion of knee

N224A Bakers cyst

N2251 Rupture of Bakers cyst - knee

N2266 Patellar tendon nontraum.rupt.

N22y4 Synovial plica of knee

N22yJ Abscess of bursa-knee

N2431 Hypertrophy of knee fat pad

N300R Acute osteomyelitis-patella

N302R Infection of patella

N310F Paget's disease-patella

N3241 Kohler's dis.(prim.patell.ctr)

N3243 Sinding-Larsen's dis.(patella)

N3270 Osteochondritis dissec-patella

N3272 Other osteochondr dissec-knee

N32z2 Osteochondritis of knee

N3373 Algodystrophy of knee

N364 Acquired genu valgum/varum

N3640 Knock knee

N364z Acquired genu valgum/varum NOS

N365 Genu recurvatum - acquired

N366 Acquired knee deformity NOS

N3660 Flexion deformity of knee

N368 Other knee deformity

N3682 Chronic instability of knee

N36yD Deformity of patella

Nyu35 [X]Other derangements/patella

Nyu36 [X]Other disorders of patella

Nyu37 [X]Other meniscus derangements

Nyu38 [X]O spontn disrptn/lig(s)knee

Nyu39 [X]Oth intrnl derangemnts/knee

Nyu3E [X]Disorder of patella, unspec

NyuA1 [X]Other bursitis of knee

OX7130B Osteoarthrosis Knee(S) /ox

R022C [D]Lump on knee

S32 Fracture of patella

S320 Closed fracture of the patella

S3200 Closed # patella transverse

S3201 Closed # patella,proximal pole

S3202 Closed # patella, distal pole

S3203 Closed # patella, vertical

S3204 Cls # patella, stellate

S321 Open fracture of the patella

S3210 Open # patella, transverse

S3211 Open # patella, proximal pole

S3212 Open # patella, distal pole

S3213 Open # patella, vertical

S3214 Open # patella, stellate

S32z Fracture of patella NOS

S46 Dislocation/subluxation knee

S460 Acute meniscal tear medial

S4600 Ac meniscal tear,med,ant horn

S4601 Ac meniscal tear,med,post horn

S4602 Ac menscl tear,med,bckt hndle

S4603 Ac meniscal tear,med,radial

S4604 Ac mnscl tr,med,periph,dtchmt

S4605 Ac mnscl tear,med,horiz clvge

S461 Acute meniscal tear lateral

S4610 Ac meniscal tear,lat,ant horn

S4611 Ac meniscal tear,lat,post horn

S4612 Ac menscl tear,lat,bckt hndle

S4613 Ac meniscal tear,lat,radial

S4614 Ac mnscl tr,lat,periph,dtchmt

S4615 Ac mnscl tear,lat,horiz clvge

S462 Other acute meniscus tear

S463 Cls trm dslctn patello-fem jt

S4630 Cls trm dslctn pat-fem jt,lat

S4631 Cls trm dslctn pat-fem jt,med

S464 Opn trm dslctn patello-fem jt

S4640 Opn trm dslctn pat-fem jt lat

S4641 Opn trm dslctn pat-fem jt,med

S465 Other cls trm dslctn knee

S4650 Cls trm dslctn knee, unspec

S4651 Cls trm dslctn knee jt, ant

S4652 Cls trm dslctn knee jt, post

S4653 Cls trm dslctn knee jt, medial

S4654 Cls trm dslctn knee jt,lateral

S4655 Cls trm dslct knee jt,rotatory

S4656 Cls trm dslctn, head fibula

S465z Cls trm dslctn knee NOS

S466 Other opn trm dslctn knee

S4660 Opn trm dslctn knee, unspec

S4661 Opn trm dslctn knee jt, ant

S4662 Opn trm dslctn knee jt, post

S4663 Opn trm dslctn knee jt, medial

S4664 Opn trm dslctn knee jt,lateral

S4665 Opn trm dslct knee jt,rotatory

S4666 Opn trm dslctn, head fibula

S466z Open dislocation knee NOS

S467 Cls trmtc sublux pat-fem jt

S4670 Cls trm sublux pat-fem jt,ltrl

S4671 Cls trm sublux pat-fem jt,med

S468 Opn trmtc sublux pat-fem jt

S4680 Opn trm sublux pat-fem jt,ltrl

S4681 Opn trm sublux pat-fem jt,med

S469 Cls trmtc sublux knee jt

S4690 Cls trmtc sublux knee jt,unsp

S4691 Cls trmtc sublux knee jt,ant

S4692 Cls trmtc sublux knee jt,post

S4693 Cls trm sublux knee jt,medial

S4694 Cls trmtc sublux knee jt,ltrl

S4695 Cls trm sublux knee jt,rotatry

S4696 Cls trmtc sublux,head fibula

S46A Opn trmtc sublux knee jt

S46A0 Opn trmtc sublux knee jt,unsp

S46A1 Opn trmtc sublux knee jt,ant

S46A2 Opn trmtc sublux knee jt,post

S46A3 Opn trm sublux knee jt,medial

S46A4 Opn trmtc sublux knee jt,ltrl

S46A5 Opn trm sublux knee jt,rotatry

S46A6 Opn trmtc sublux,head fibula

S46B Tear/articulr cart/knee,currnt

S46C Inj/multipl structures of knee

S46D Recurrent subluxation, patella

S46z Dislocation of knee NOS

S4F #-dslc/subluxation knee

S4F0 Closed #-dslc, knee joint

S4F1 Open #-dslc, knee joint

S4F2 Closed #-sublux, knee joint

S4F3 Open #-sublux, knee joint

S4F4 Cls #-dslc,patello-fem jt

S4F5 Open #-dslc,patello-fem jt

S4F6 Cls #-sublux,patello-fem jt

S4F7 Open #-sublux,patello-fem jt

S534 Sprain patellar tendon

S54 Knee sprain

S540 Sprn/prt tr,knee,lat coll lgmt

S5400 Sprn,knee jt,lat collat lgmt

S5401 Part tear,knee,lat collat lgmt

S541 Sprain med.collateral lig.knee

S5410 Sprn,knee jt,medial collat

S5411 Part tear,knee,mdl collat lgmt

S542 Sprain cruciate ligament knee

S5421 Part tr,knee,ant cruciate lgmt

S5422 Prt tr,knee,post cruciate lgmt

S54w Other specified knee sprain

S54y Knee sprain NOS

S5C Complete tear, knee ligament

S5C0 Cmplt tr,knee,lat collat lgmt

S5C1 Cmplt tr,knee,mdl collat lgmt

S5C2 Cmp tr,knee,post cruciate lgmt

S5C3 Cmpl tr,knee,ant cruciate lgmt

S5Cy Cmplt tr,other knee lgmt

S5Cz Cmplt tr,knee lgmt NOS

S5K Open division ligament knee

S5K0 Opn dvsn lat collat lgmt knee

S5K1 Opn dvsn mdl collat lgmt knee

S5K2 Opn dvs post cruciate lgm knee

S5K3 Opn dvs ant cruciate lgmt knee

S5Ky Open division other knee lgmt

S5Kz Open division knee lgmt NOS

S5U2 Rupture patellar tendon

SA100 Open wound of knee

SA110 Open wound knee+complication

SD6y2 SuperficialinjuryofkneeNOS

SE41 Bruise - knee/lower leg

SE411 Contusion knee

SF311 Crush injury knee

SK170 Other knee injury

Syu84 [X]Sprn/str oth unsp part knee

# Generalised / Widespread musculoskeletal problems

**Code Term**

14G H/O: musculoskeletal disease

1DCC Aching muscles

N000 Systemic lupus erythematosus

N0000 Disseminated lupus erythemat.

N000z Systemic lupus erythematosus NOS

N001 Systemic sclerosis

N0011 CREST syndrome

N004 Polymyositis

N006 Antiphospholipid syndrome

N0310 Arthropathy in ulcerative colitis

N038 Reactive arthropathies

N04 Rheumatoid arthritis+similar

N040 Rheumatoid arthritis NOS

N040P Seronegative rheumatoid arthritis

N040S Rheumat arthr - multiple joint

N040T Flare of rheumatoid arthritis

N041 Feltys syndrome

N043 Stills disease - juvenile R.A

N0431 Acute polyartic.juvenile R.A.

N043z Juvenile rheumatoid arthritis NOS

N045 Other juvenile arthritis

N0455 Juvenile rheumatoid arthritis

N04X Seroposit rheum arthr unsp

N04y1 Sero negative polyarthritis

N04y2 Adult-onset Stills disease

N04z Inflammatory polyarthropathy NOS

N050 Generalised osteoarthritis - OA

N0500 Generalised OA-site unspecif.

N0502 Generalised OA-multiple sites

N0504 Primary generalized osteoarthrosis

N050z Generalised osteoarthritis NOS

N065 Unspecified polyarthropathy

N0659 Unsp.polyarthr.-multiple site

N065A Generalised arthritis

N065z Polyarthritis

N06z9 Arthropathy NOS of multiple sites

N0949 Arthralgia of multiple joints

N0959 Multiple stiff joints

N096 Musculoskeletal pain - joints

N0969 Other joint sympt.-multip.site

N0975 Walking difficulty-multip.site

N099A Multiple clicking joints

N09z9 Joint disord.NOS-multiple site

N0z Arthropathies NOS

N20 Polymyalgia

N200 Gnt cell arter+polymyalg rheum

N23 Fascia disorders

N232 Muscle wasting/atrophy NEC

N2331 Immobility syndrome

N235 Hypermobility syndrome

N237 Other fibromatoses

N239 Fibromyalgia

N24 Other soft tissue disorders

N2400 Rheumatism NOS - multiple

N2401 Fibrositis unspecified

N248 Fibromyalgia

N24z Polyalgia

N310 Pagets disease of bone

N310z Pagets disease NOS

N312 Hypertroph.pulm.osteoarthrop.

N33 Other bone/cartilage disorders

N330 Osteoporosis

N3300 Osteoporosis unspecified

N3301 Senile osteoporosis

N3302 Postmenopausal osteoporosis

N3303 Idiopathic osteoporosis

N3304 Dissuse osteoporosis

N3305 Drug-induced osteoporosis

N330A Osteoporosis in endocr disord

N330z Osteoporosis NOS

N331M Fragility # unsp osteoporosis

N337 Reflex sympathetic dystrophy

N33z5 Relapsing polychondritis

N33zz Bone or cartilage disorders NOS

N36y2 Deformity of bone

Nyu1 [X]Inflammatory polyarthropathies

Nyu11 [X]O sero+ve rheumat arthritis

Nyu1A [X]Oth spcfc arthropathiesNEC

Nyu20 [X]Other polyarthrosis

Nyu4 [X]Systmc connctv tis disordrs

Nyu8 [X]Disorders of muscles

Nyu84 [X]Muscle wasting and atrophy NEC

NyuB8 [X]Unsp osteopor + pathol frac

NyuBC [X]Osteopenia

OX7120DA Juvenile Arthritis /ox

R00z2 [D]General aches and pains

R01 [D]Musculoskeletal symptoms

R027 [D]Spontaneous ecchymoses

S3z Fractures

S5z Sprains and strains NOS

SDz Superficial injuries NOS

SE44 Contusionlwr limbmlti sites

SE4y Contusion multiple sites NEC

SK1x Other multiple injuries

SR1z0 [X]Clsd multiple fracts unspec
